# Flexible bioelectronic device fabricated by conductive polymer–based living material

**DOI:** 10.1126/sciadv.abo1458

**Published:** 2022-06-22

**Authors:** Zenghao Wang, Haotian Bai, Wen Yu, Zhiqiang Gao, Weijian Chen, Zhiwen Yang, Chuanwei Zhu, Yiming Huang, Fengting Lv, Shu Wang

**Affiliations:** 1 Beijing National Laboratory for Molecular Sciences, Key Laboratory of Organic Solids, Institute of Chemistry, Chinese Academy of Sciences, Beijing 100910, P. R. China.; 2 College of Chemistry, University of Chinese Academy of Sciences, Beijing 100049, P. R. China.

## Abstract

Living materials are worked as an inside collaborative system that could naturally respond to changing environmental conditions. The regulation of bioelectronic processes in living materials could be effective for collecting biological signals and detecting biomarkers. Here, we constructed a living material with conjugated polymers poly[3-(3′-*N*,*N*,*N*-triethylamino-1′-propyloxy)-4-methyl-2,5-thiophene chloride] (PMNT) and *Shewanella oneidensis* MR-1 biofilm. In addition, the living material was integrated as a flexible bioelectronic device for lactate detection in physiological fluids (sweat, urine, and plasma). Owing to the electroconductivity of conjugated polymers, PMNT could optimize the bioelectronic process in the living material. The collected electrical signal could be wirelessly transferred to a portable smartphone for reading and analyzing. Because lactate is also a biomarker for cancer treatment, the flexible bioelectronic device was further used to detect and count the cancer cells. The proof of the bioelectronic device using conductive polymer–based living material exhibits promising applications in the next-generation personal health monitoring systems.

## Teaser

A living material of *S. oneidensis* biofilm and conductive polymer PMNT is used for lactate detection and tumor cell counting.

## INTRODUCTION

Owing to the integration of disciplines between materials science and synthetic biology, the thriving living materials have evolved into a distinct field of study recently (*1*, *2*). Living materials are a type of biohybrid material consisting of living elements and nonliving components. The living elements including bacteria, cells, fungi, and algae can endow materials with distinct functional properties, such as environmental bioremediation, in situ biosensing, biomolecular synthesis, and biomedical therapeutics (*3*). The nonliving components can protect living elements from extreme environments such as low temperature, high salinity, and water shortages (*4*). Benefiting from the combination of their respective advantages, living materials could react to the changing environmental conditions and have useful function (*5*, *6*). Bacteria could respond to various environmental stimulations of molecular effectors, pH, salinity, and temperature (*7*–*9*), so it is an ideal candidate for establishing living materials. Many different living materials based on *Bacillus subtilis* (*10*), *Escherichia coli* (*11*), and *Lactococcus lactis* (*12*) have been used for biocatalysis (*13*), biosensors (*14*), biomedical applications (*15*), environmental remediation (*16*), etc. (*17*). The bioelectronic devices that transduce biological interactions into amplified electronic signals have been widely used in wearable and implantable apparatus (*18*). Integration with the advantages of living materials could provide an innovative strategy in the development of bioelectronic devices. Biofilms are structured bacterial communities on various surfaces that are formed by the synthesis and secretion process of a cohesive and protective extracellular matrix (*19*). As the protecting clothing, the biofilm can protect the biological activities of organisms from multiple extreme environments. The clear macrostructure and stable biofunction of bacteria make the biofilm more suitable for constructing living materials. Besides, there are abundant interactions between the living and nonliving elements. The integration of living and nonliving components is vital for creating a coordinative environment to accommodate rich interactions. For example, the study and optimization of the interaction between living and nonliving components are helpful to realize the conversion and amplification of biological signals (*20*). Compared to output signals of protein and fluorescence, the slight electrical signal is more sensitive and can be transmitted precisely (*21*). Hence, the study and regulation of bioelectronic processes in living materials are promising for collecting the required biological signals and determining biomarkers. The revolution based on living materials and bioelectronics theory aims to subvert the traditional developing strategy of responsive material and dynamic biomedical devices.

Conjugated polymers (CPs) are characterized by a delocalized electronic structure that allows electron transfer along the whole backbone. Thus, they have been widely used in electronic and sensor devices (*22*). Water-soluble CPs (WSCPs) are composed of π-conjugated backbones and charged side chains (*23*, *24*). For their excellent water solubility and biocompatibility, WSCPs have been widely used in the field of bioelectronics and biosensing (*25*–*31*). In addition, WSCPs can regulate the bioelectronic processes of microorganisms for realizing novel functions. For example, cationic polythiophene derivative {poly[3-(3′-*N*,*N*,*N*-triethylamino-1′-propyloxy)-4-methyl-2,5-thiophene chloride] (PMNT)} could increase the maximum current and power generated by the *Shewanella oneidensis* MR-1 (*32*). As a well-characterized model electrogenic microorganism, (*S. oneidensis*) MR-1 could oxidize lactate to generate electrons in its respiration process (*33*–*35*). Lactate is a major analyte in bioprocess engineering, sports medicine, and clinical care unit (*36*), and it has also been proved to be a reliable biomarker for cancer treatment (*37*). It is supposed that CPs could be used as a nonliving component to fabricate a living material, in which *S. oneidensis* MR-1 is the living element. By regulating the bioelectronic processes, CPs would enhance the electron transport of *S. oneidensis* MR-1 for improved sensitivity.

**Fig. 1.**
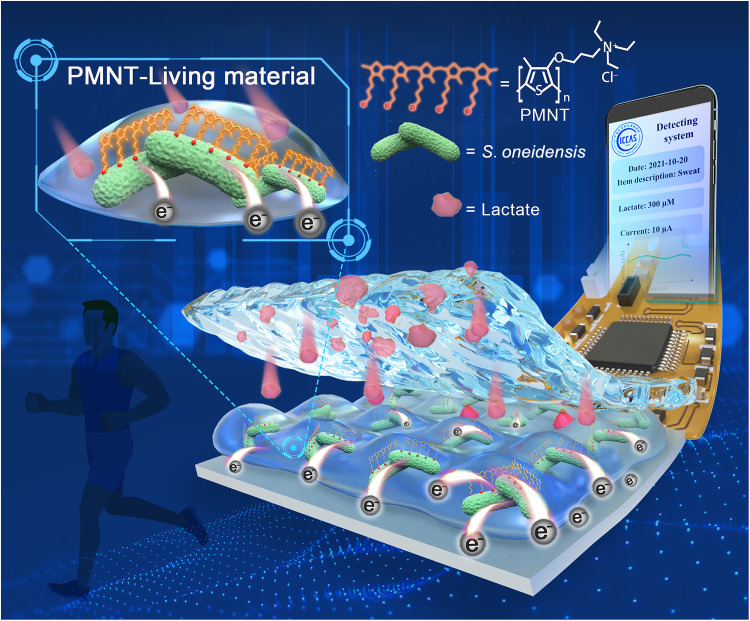
A bioelectronic device fabricated by living material for lactate detection. Schematic diagram of PMNT-Living material and bioelectronic device for monitoring lactate in physiological fluids (sweat, urine, and plasma) and counting the number of cancer cells.

In this work, we fabricated a PMNT-Living material that was composed of cationic CP PMNT and *S. oneidensis* MR-1 biofilm by stable electrostatic interaction. Meanwhile, it was integrated with bioelectronic devices for wireless monitoring the lactate in physiological fluids of sweat, urine and plasma (Fig. 1). In the living material of PMNT/*S. oneidensis* biofilm, the *S. oneidensis* MR-1 could oxidize lactate to yield electrons due to its natural respiration process, and PMNT could promote biofilm formation and enhance the electron transfer rate based on the characterized electroconductivity of CPs. The collected electrical signal could be wirelessly transferred to a portable smartphone for reading and analyzing. Attributing to the optimized bioelectronic process, the flexible bioelectronic device that integrated this PMNT-Living material had a lactate detection limit of 78 μM. Lactate is also a representative biomarker for cancer treatment, so the constructed flexible bioelectronic device could realize the detection and counting of cancer cells. This strategy will further advance the development of living materials. The flexible bioelectronic devices based on living materials are expected to promote the next-generation personal health monitoring systems.

## RESULTS

**Fig. 2.**
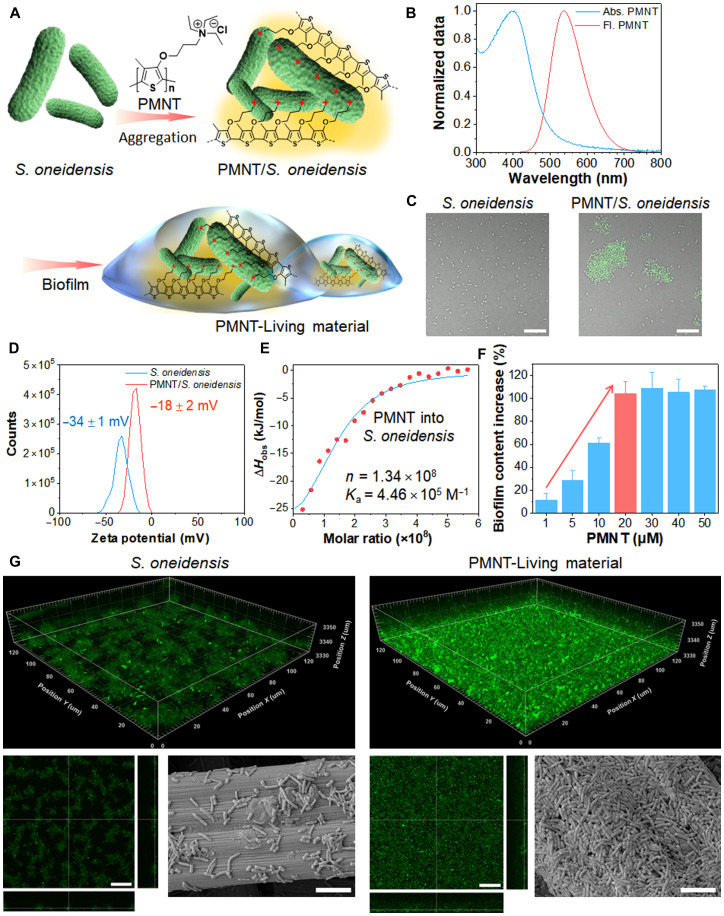
The construction and characterization of PMNT-Living material. (**A**) Schematic carton diagram for constructing PMNT-Living material of PMNT/*S. oneidensis* biofilm. (**B**) Spectral characterization of PMNT. (**C**) CLSM images of *S. oneidensis* in the absence and presence of PMNT. The fluorescence of PMNT is highlighted in green. Scale bars, 10 μm. (**D**) Zeta potentials of *S. oneidensis* before and after treating with PMNT. (**E**) ITC curve of the titration of PMNT into *S. oneidensis* and the thermodynamic parameters of the binding between PMNT and *S. oneidensis* derived from the ITC curve. (**F**) The biofilm content increase ratio (PMNT/*S. oneidensis*:*S. oneidensis*) was obtained using PMNT concentrations of 0 to 50 μM. (**G**) CLSM and SEM images of the *S. oneidensis* and the PMNT/*S. oneidensis* biofilm. Scale bars, 20 μm (left) and 5 μm (right).

For constructing the PMNT-Living material of PMNT/*S. oneidensis* biofilm (Fig. 2A), the confocal laser scanning microscopy (CLSM), zeta potential, and isothermal titration calorimetry (ITC) were first carried out to investigate the interaction between PMNT and bacteria. Because of the specific photophysical property of PMNT (Fig. 2B), CLSM imaging was used to observe the biological action directly. As shown in Fig. 2C, the bulked green signals in CLSM images demonstrated that the PMNT could well stain on the *S. oneidensis* MR-1 and promote bacterial aggregation. For the control group, the pristine bacteria without PMNT were dispersed uniformly and did not exhibit the tendency to aggregate. The zeta potential of *S. oneidensis* MR-1 was positively shifted from −34 ± 1 mV to −18 ± 2 mV only after incubating with PMNT (Fig. 2D). As a Gram-negative bacterium, the electronegative outer membrane of *S. oneidensis* MR-1 consists of phospholipids, lipopolysaccharides, integral membrane proteins, and lipoproteins (*8*). Hence, the cationic CPs with quaternary ammonium groups could attach and insert the bacterial membrane via electrostatic and hydrophobic interactions. It has been reported that the hydrophobic interaction is hard to affect the zeta potential of bacteria, yet the electrostatic interaction would cause a positive potential shift (*38*). The remarkable potential change of the PMNT/*S. oneidensis* group was attributed to the electrostatic interaction. It was also consistent with the following ITC analysis. As shown in Fig. 2E, the observed enthalpy (Δ*H*_obs_) reached the maximum exothermic value (about −25 kJ/mol) when PMNT was added into the bacterial solution. Along with adding PMNT, the Δ*H*_obs_ was gradually returned to zero. In this case, the number (*n*) of PMNT associated with a single *S. oneidensis* MR-1 was around 10^8^, and the binding affinity between them was described by the calculated bind constant (*K*_a_: 4.46 × 10^5^ M^−1^). These thermodynamic changes demonstrated that the interaction between the PMNT and bacteria was an exothermic process, which was mainly contributed by the electrostatic binding behavior (*27*). It was the strong electrostatic interaction that brought the above staining and aggregating characters of PMNT/*S. oneidensis*. Moreover, the bacterial aggregation tended to form microcolony, which is an essential step for biofilm formation (*9*). The normal crystal violet staining was subsequently used to characterize the favorable effect of PMNT on promoting the *S. oneidensis* MR-1 biofilm formation quantitatively (*39*). As shown in Fig. 2F, the content of *S. oneidensis* MR-1 biofilm was gradually increased along with the improved PMNT from 1 to 20 μM. Compared with the free *S. oneidensis* MR-1 group, only 1 μM PMNT could lead to about a 10% increase of *S. oneidensis* MR-1 biofilm, and 20 μM PMNT could double the amount. Then, the biofilm content almost stopped further growing even with 50 μM PMNT. The reached maximum was the selected optimum condition of PMNT for forming *S. oneidensis* MR-1 biofilm. The result was also evidenced that the PMNT/*S. oneidensis* aggregation was a benefit for biofilm formation. The good biocompatibility of PMNT toward *S. oneidensis* MR-1 was measured and showed that even 70 μM PMNT could not influence the bacterial viability (figs. S1 and S2). When the concentration of PMNT increased to 150 μM, the growth of *S. oneidensis* MR-1 was inhibited (inhibition rate of 20%) (fig. S2). Because of the improved gathering effect and biofilm communication, the PMNT was an essential factor for constructing the designed PMNT-Living material. The morphologic information of the prepared PMNT-Living material of PMNT/*S. oneidensis* biofilm was investigated and visualized by CLSM and scanning electron microscopy (SEM) imaging. The standard Live/Dead viability analysis of the propidium iodide (PI) and SYTO 9 was used to depict the microbial metabolism status and identify the biofilm, where all bacterial cells were revealed as distinct green fluorescence signals from SYTO 9, and the dead bacteria were stained with red fluorescence signals from PI. As shown in Fig. 2G, the green fluorescence from PMNT/*S. oneidensis* biofilm was significantly stronger than the control group of *S. oneidensis* MR-1. The thickness of PMNT/*S. oneidensis* biofilm was around 20 μm, which was also denser and thicker than the control group of fewer than 10 μm. It suggested that the number of bacteria in the PMNT/*S. oneidensis* biofilm was much larger than the control group. In addition, the weak red fluorescence in both groups suggested that only a few bacteria were dead in the biofilm (fig. S3). The more obvious red signals in the PMNT/*S. oneidensis* group were due to the hypoxic environment of mature biofilm that could cause the natural death of bacteria inside. It verified that the biofilm of PMNT/*S. oneidensis* was much more complete than the *S. oneidensis* MR-1 alone for the same incubation time. As the details shown in SEM images, the pristine bacteria were exiguous and sporadic on the substrate, and it proved that the biofilm had not formed yet. In comparison, a mass of microbial cells pretreated with PMNT was fully covered and closely packed on the substrate. The clear edges and the surface integrity of *S. oneidensis* MR-1 graphically presented the morphology and viability of the formed PMNT/*S. oneidensis* biofilms. All these phenomena demonstrated that the PMNT could boost the formation of *S. oneidensis* MR-1 biofilm. The obtained PMNT/*S. oneidensis* biofilm would act as the expected PMNT-Living material for the following investigation and application.

**Fig. 3.**
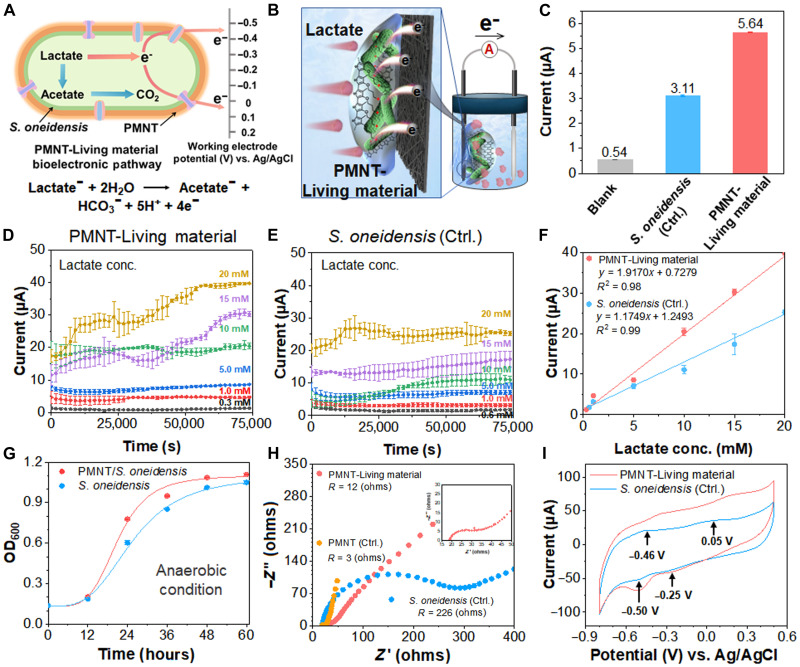
The construction of the detecting system and the electrochemical measurements. (**A**) Schematic diagram of electron transport pathway in PMNT-Living material. (**B**) Schematic carton diagram for detecting system of PMNT-Living material with improved electrogenic property. (**C**) The magnitude of currents generated by the detecting system in the absence of different elements. (**D**) The current curves are generated by the detecting system of PMNT-Living material. Each line represents the average of three biological replicates, with SE shown in error bars (*n* = 3). (**E**) The current curves generated by the detecting system of the *S. oneidensis* MR-1. Each line represents the average of three biological replicates, with SE shown in error bars (*n* = 3). (**F**) The calibration plots of (D) and (E). Each point represents the average of three biological replicates, with SE shown in error bars (*n* = 3). The error bars corresponding to different concentrations of PMNT-Living materials are 0.11, 0.26, 0.21, 0.89, 0.74, and 0.30, respectively. The error bars corresponding to different concentrations of *S. oneidensis* are 0.20, 0.44, 0.60, 1.0, 2.5, and 0.68, respectively. (**G**) The growth curves of *S. oneidensis* MR-1 in the absence and presence of PMNT under anaerobic condition. Each line represents the average of three technical replicates, with SE shown in error bars (*n* = 3). (**H**) The Nyquist curves of PMNT, *S. oneidensis* MR-1, and PMNT-Living material (inset). (**I**) CV curves of the *S. oneidensis* MR-1 and the PMNT-Living material.

*S. oneidensis* MR-1 is a well-studied facultative anaerobic exoelectrogens, which could preferentially use lactate as the carbon source and the electron donor to produce bioelectricity. Electron transport from *S. oneidensis* MR-1 includes direct electron transport through c-type cytochromes and indirect electron transport through electron shuttles such as flavin (Fig. 3A) (*40*). Subsequently, a bioelectronic device that could evaluate the generated bioelectricity was set up for detecting lactate, where the PMNT-Living material and MnO_2_ (*41*) were used as the anode and cathode, respectively (Fig. 3B and fig. S4). Under anaerobic conditions, the *S. oneidensis* MR-1 could generate 3.11 μA of current by consuming lactate and extracellular electron transfer (EET) process. In comparison, the current of PMNT-Living material was increased to 5.64 μA under the same condition (Fig. 3C). The constructed PMNT-Living material owned better response capability toward lactate than the single *S. oneidensis* MR-1. Then, a gradient of lactate was measured by the PMNT-Living material and *S. oneidensis* MR-1 systems, respectively, and a series of current curves were recorded between 0.0 and 75000.0 s. The current responses of *S. oneidensis* MR-1 were closely related to the bacterial physiological state and the quantity of the bacteria, so the current curves varied over time and were uneven as shown in Fig. 3 (D and E). As shown in Fig. 3D, only 300 μM lactate could launch the bioelectronic device of PMNT-Living material, and the current intensity increased with the concentration of lactate. Although a similar strengthening tendency appeared in the *S. oneidensis* MR-1 group, the initial concentration had doubled to 600 μM (Fig. 3E). The current responses of *S. oneidensis* MR-1 were closely related to the dynamic physiological state and the quantity of the bacteria, which may cause a moderate fluctuation of the current as shown in Fig. 3 (D and E). Meanwhile, the produced bioelectricity of the two systems could reach a steady state, so the growth of corresponding current curves would flatten out eventually. In addition, the corresponding calibration plots displayed good linearity between the lactate concentration and the produced current intensity (Fig. 3F). Moreover, the PMNT-Living material produced a higher magnitude of currents than the control group of *S. oneidensis* MR-1 at the corresponding concentrations of lactate. On the basis of the SD of the response and the slope, we could estimate that the lactate detection limit of PMNT-Living material system was 78 μM, while that of the *S. oneidensis* MR-1 was higher to 128 μM (signal/noise = 3) (*42*). Considering that the detected current intensity was directly associated with the amount of released electron from *S. oneidensis* MR-1 and the conductivity, the bacterial growth curve, electrochemical impedance spectroscopy (EIS), and cyclic voltammetry (CV) were conducted and investigated the mechanism of the detection improved performance. As shown in Fig. 3G and fig. S5, the anaerobic growth curve of PMNT/*S. oneidensis* could reach the stationary phase for the 36-hour incubation. For the *S. oneidensis* MR-1 group, it took an extra 12 hours to reach a similar state. Because the PMNT had a delocalized electronic structure that allows electron transfer along the whole backbone, it was hypothesized that the PMNT could enhance the characteristic EET and could achieve the effect of acceleration. The electrochemical properties of *S. oneidensis* MR-1 in the presence and absence of PMNT were measured by EIS and CV, and the Nyquist plots consisted of a semicircle and a straight line. As shown in Fig. 3H, the charge transfer resistance (*R*_ct_) of the PMNT-Living material was 12 ohms, which was much less than that of the *S. oneidensis* MR-1 alone (226 ohms), and the PMNT exhibited the lowest *R*_ct_ of 3 ohms. Because the value of *R*_ct_ was inversely proportional to electron transfer rate (*43*), it suggested that the electron transfer rate of PMNT-Living material was faster than that of the control group, and the results could be additionally attributed to the good conductivity of PMNT. In addition, the CV results echoed the positive effect of PMNT for electron transfer (Fig. 3I). The free *S. oneidensis* MR-1 had two pairs of redox peaks: one is an oxidation peak at around 0.05 V and a reduction peak at around −0.25 V, and the other one is an oxidation peak at around −0.46 V and a reduction peak at around −0.50 V (*40*). The two pairs of redox peaks could be exactly ranged to the c-type cytochromes and flavins distributed on the bacterial membrane (*32*). For the PMNT-Living material group, redox peaks of PMNT and *S. oneidensis* MR-1 appeared simultaneously, the redox potential was changed, and the redox peak was increased. It demonstrated the efficient direct electron transfer from the PMNT to membrane proteins in the well-combined PMNT/*S. oneidensis* biofilm. These results verified that PMNT improved the bioelectronic performances of the PMNT-Lving material, so the constructed bioelectronic device incorporated with the PMNT-Living material is advanced in lactate monitoring and detection.

**Fig. 4.**
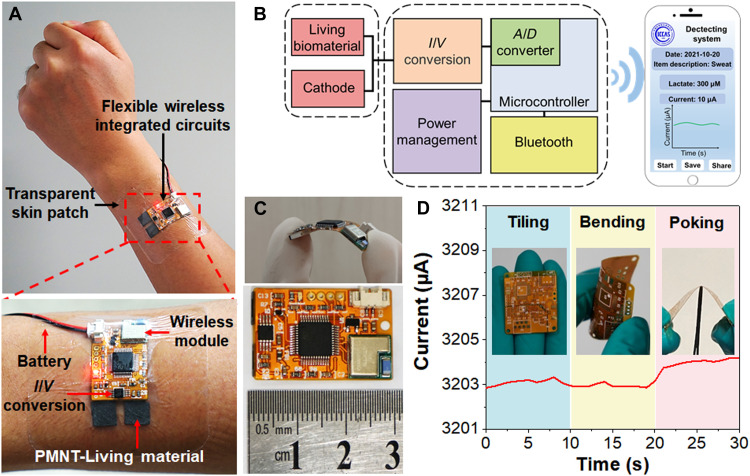
The flexible bioelectronic device for the detection of lactate. (**A**) A photograph of a flexible wireless lactate-monitoring bioelectronic device. (Magnified view) Components of the flexible wireless lactate-monitoring chip. (**B**) Simplified block diagram of the bioelectronic device. (**C**) Photograph of the device, next to a ruler. (**D**) The current of a fully stretchable substrate under sequential no strain, bending, and poking with a sharp object.

**Fig. 5.**
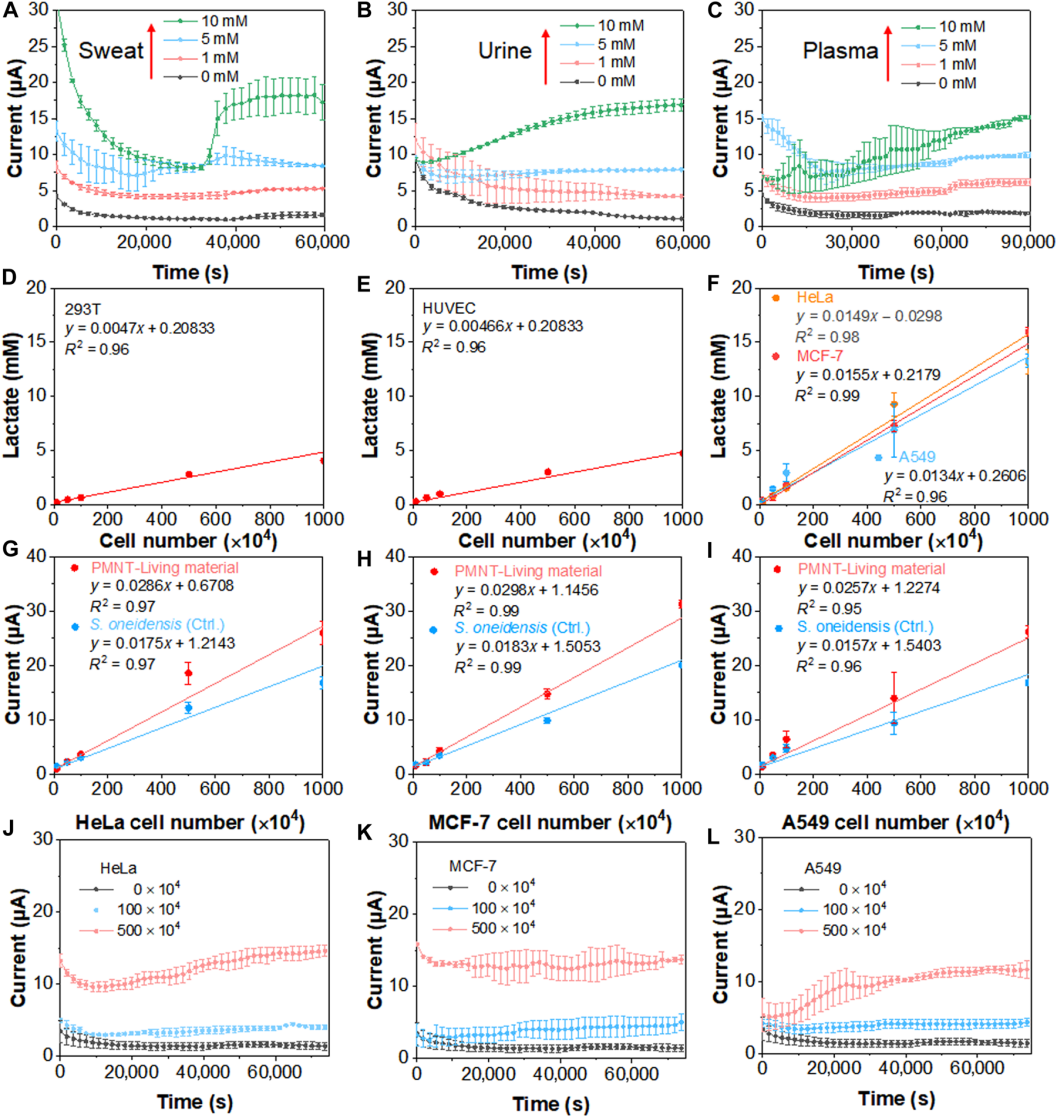
The flexible bioelectronic device for the detection and counting of cancer cells. Current responses generated by the bioelectronic device with the addition of lactate in sweat (**A**), urine (**B**), and plasma (**C**). Each line represents the average of three biological replicates, with SE shown in error bars (*n* = 3). The calibration plots of lactate and cell count of 293T (**D**) and human umbilical cord endothelial cell (HUVEC) (**E**) (*n* = 3). (**F**) The calibration plots of lactate and cell count of HeLa, MCF-7, and A549 (*n* = 3) (**G** to **I**). The calibration plots of current and cell count of HeLa, MCF-7, and A549, respectively (*n* = 3). Current responses generated by the bioelectronic device with the addition of HeLa (**J**), MCF-7 (**K**), and A549 (**L**) cells. Each line represents the average of three biological replicates, with SE shown in error bars (*n* = 3).

The high-performance uniformity of this fabrication lactate detection system allowed us to develop intrinsically basic circuit elements (*44*). The bioelectronic device of PMNT-Living material was further integrated with a flexible chip, and the Bluetooth technology was exploited to monitor the concentration of lactate in the wireless. To support the potential of long-term interaction and tracking of the lactate produced by the human body, the bioelectronic device was conformably attached to the skin by covering with a transparent skin patch (Fig. 4A). The functional components were presented in Fig. 4B, an external set of battery delivered power to the device, and sequentially activated the current-voltage relation (*I*/*V*) conversion, the analog/digital (*A*/*D*) converter, and the Bluetooth hardware for wireless transmission. The output parameter was lastly transmitted to the smartphone of a customer for recording and analysis. Besides, the small size of the circuit elements was only 3 cm, which was also promising for the applications (Fig. 4C). The output current of the circuit elements under different operations of tilling, bending, or poking was recorded as well. The results in Fig. 4D verified that the current generated by the flexible device could maintain stable performance under extreme conditions. In addition, the bioelectronic device was used to detect the lactate in body fluids. As shown in Fig. 5 (A to C), the magnitude of currents in a steady state was all positively correlated with the concentration of lactate. With the addition of 0, 1, 5, and 10 mM lactate in sweat, urine, and plasma, the response current increased with time and eventually reached a steady state. In addition, the current responses of *S. oneidensis* MR-1 are closely related to the bacterial physiological state and the quantity of the bacteria, so the shape and position of the current curves may vary over time and across experiments. For the control group (without PMNT), the response current also increased with time and eventually reached a steady state. The magnitude of currents in the control group (fig. S6) was much less than that in the experimental group (Fig. 5, A to C). The bioelectronic devices without PMNT can hardly distinguish the lactate concentration of 1 mM in sweat and urine. These results demonstrated that such a bioelectronic device was a good candidate for performing long-term, continuous, convenient, and accurate detection of lactate produced by the human body.

According to the Warburg effect, cancer cells tend to convert glucose to lactate even under adequate oxygen conditions (*45*). The accumulated lactate in the tumor microenvironment could regulate the development, maintenance, and metastasis of tumors, and lactate has been proved to be a reliable biomarker for cancer. Subsequently, the lactate produced by normal cells and cancer cells was investigated (*42*). The HeLa, MCF-7, and A549 were selected as the cancer cells lines, while the 293T and human umbilical cord endothelial cell (HUVEC) were selected as the normal cell lines. As displayed in Fig. 5 (D to F), the corresponding calibration plots displayed good linearity between the concentration of lactate and cell count in all the cell lines. As the reliable biomarker for cancer, lactate concentrations produced by normal cells of both 293T and HUVEC were much lower than those produced by cancer cells (HeLa, MCF-7, and A549). These results demonstrated that the number of cancer cells could be determined by measuring the concentration of lactate in the tumor microenvironment. Then, we further investigated the correlation between the magnitude of currents and the cancer cell count. As shown in Fig. 5 (G to I), the corresponding currents in PMNT-Living material and *S. oneidensis* MR-1 group presented a clear linear correlation with the cell count in different cancer cell lines of HeLa, MCF-7, and A549. In addition, the lower detection limit (LDL) of the PMNT-Living material system was lower than that of the *S. oneidensis* MR-1 group. The current values were calculated by substituting 0.3 mM (PMNT-Living material) and 0.6 mM (*S. oneidensis*) into Fig. 3F. Then, the calculated currents were substituted into Fig. 5 (G to I) to calculate the number of corresponding cells. The LDLs of PMNT-Living material system toward HeLa, MCF-7, and A549 cells were 22 × 10^4^, 5.3 × 10^4^, and 2.9 × 10^4^, while those of *S. oneidensis* MR-1 group were 42 × 10^4^, 25 × 10^4^, and 26 × 10^4^, respectively. As mentioned above, it was because that PMNT could accelerate the formation and the electron transfer in the living material of PMNT/*S. oneidensis*, increasing the generated current of the bioelectronic device. Subsequently, the cancer cell numbers were performed in the aforesaid bioelectronic device and detection system (Fig. 4A). As shown in Fig. 5 (J to L), the current generated by the bioelectronic device increased with time and eventually reached a steady state. The magnitude of current in a steady state was positively correlated with the cell number. Errors of the measurement of the three cancer cells were shown in table S1. The relative errors of 100 × 10^4^ and 500 × 10^4^ HeLa cells were 7.6 and 2.7%, respectively. The corresponding data for MCF-7 and A549 were 5.6 and 6.8%, and 7.4 and 9.9%, respectively. These results demonstrated that the fabricated bioelectronic device could sensitively detect the lactate concentration in situ and accurately measure the number of cancer cells.

## DISCUSSION

We have constructed a PMNT-Living material that is composed of a cationic CP PMNT and *S. oneidensis* MR-1 biofilm through electrostatic interactions. The living material is then integrated as a flexible bioelectronic device for lactate detection in physiological fluids (sweat, urine, and plasma). The PMNT contributed to the formation of biofilm of *S. oneidensis* MR-1 and enhancing the electron transfer rate due to the electroconductivity of CPs. Hence, *S. oneidensis* MR-1 owned the improved electrogenic ability, and PMNT-Living material owned the accelerated oxidization process from lactate to electrons. All the collected electrical signals by the flexible bioelectronic device could be further wirelessly transferred to a portable smartphone for reading and analyzing. In addition, lactate is a well-recognized tumor marker, so the constructed flexible bioelectronic device successfully realized the cancer cell detection and counting. As a proof of concept, this work performed the promising application of living materials in physiological and clinical investigations, and it further paved the way to develop flexible devices in the next-generation personal health monitoring systems.

## MATERIALS AND METHODS

### Materials and instruments

Cationic CP (PMNT) was synthesized on the basis of the literature. *S. oneidensis* MR-1 was purchased from Marine Culture Collection of China. The liquid medium and double-distilled water (ddH_2_O) were autoclaved at 120°C for 20 min. Phosphate-buffered saline (PBS) was purchased from Hyclone (Logan, UT). A BacLight Live/Dead viability kit was purchased from Molecular Probes (Eugine, OR). Toray carbon paper (TGP-H-090) was purchased from Shanghai Chuxi Industrial Co. Ltd. The 96-well cell culture plates were obtained from Nalge Nunc International (Rochester, NY). The OD_600_ (optical density at 600 nm) and OD_590_ of *S. oneidensis* MR-1 were read on a microplate reader (Bio-Tek Synergy HT, USA). Zeta potentials were determined using a Nano ZS90 (Malvern, UK). CLSM characterization was made with a confocal laser scanning biological microscope (FV1000-IX81, Olympus, Japan). SEM images were taken from Hitachi S-4800 SEM. EIS and CV were carried out using the Autolab PGATAT302N (Metrohm, Switzerland) and Shanghai Chenhua electrochemical workstation, respectively. The current-time curves were measured using Keithley 2450. The electrodeposition was performed using the Autolab PGATAT302N (Metrohm, Switzerland). The sweat, urine, and plasma were purchased from Beijing Huizhi Taikang Co. Ltd. All other chemicals were of analytical reagent grade and used as purchased without further purification.

### The aerobic and anaerobic growth curves of *S. oneidensis* MR-1

The single colony was transferred from LB solid agar plate to 15-ml LB liquid medium. The strain was incubated overnight at 30°C. Five milliliters of bacterial solution after overnight culture was centrifuged at 7100 rpm for 3 min, and then it was diluted by PBS (10 × 10^−3^ M, pH 7.4) to adjust its OD_600_ = 1. The cultures were then diluted 1:1000 into fresh medium with or without 20 μM PMNT. Two hundred microliters of inoculation medium was put into the 96-well plates. OD_600_ was recorded every 1 hour at 30°C (*32*). The experiment was repeated three times, and the curve was drawn from the average. The single colony was transferred from LB solid agar plate to 15-ml LB liquid medium. The strain was incubated overnight at 30°C. The bacterial solution after overnight culture was centrifuged at 7100 rpm for 3 min and washed with PBS twice, and then it was diluted by LB liquid medium to adjust its OD_600_ = 1.0. Twenty milliliters of the inoculation buffer was put into sealed cell, with carbon paper as the anode and MnO_2_ as the cathode. The sealed cell was pumped with nitrogen to remove oxygen. The experimental group added 20 μM PMNT, and the control group did not add PMNT. OD_600_ was recorded every 12 hours at 30°C. The experiment was repeated three times, and the curve was drawn from the average.

### CLSM characterization of *S. oneidensis* MR-1 incubated with PMNT

The single colony was transferred from LB solid agar plate to 15-ml LB liquid medium. The strain was incubated overnight at 30°C. The bacterial solution after overnight culture was centrifuged at 7100 rpm for 3 min and washed with PBS twice. After centrifugation, the bacteria were suspended in PBS, and the experimental group was added with 20 μM PMNT incubated at 30°C for 15 min. After incubation, the mixture was centrifuged at 7100 rpm for 3 min to remove the unbound PMNT. The mixture was cleaned once with PBS and then mounted on a glass slide with a cover slide on top and examined by confocal scanning laser microscopy with a 488-nm laser (FV5-LAMAR). The fluorescence of PMNT was green (*32*).

### Zeta potential measurements

After incubated with 20 μM PMNT at 30°C for 15 min, the mixture was centrifuged at 7100 rpm for 5 min to remove the unbound PMNT and washed with ddH_2_O twice. After centrifugation at 7100 rpm for 5 min, the supernatant was removed and the precipitated pellets were suspended in ddH_2_O again (*32*). The suspension was stored on ice and measured by zeta potential. As the control group, bacteria (without PMNT) were also cultured under exactly the same conditions.

### Isothermal titration calorimetry

The calorimetric measurements were conducted on a MicroCal iTC200. The sample cells were initially loaded with 600-μl PBS or bacterial PBS solution (OD_600_ = 1.0). The concentrated PMNT solution was injected consecutively into the stirred sample cell in portions of 10 μl via a 500-μl Hamilton syringe controlled by a 612 Thermometric Lund pump until the interaction progress was completed. The system was stirred at 90 rpm with a gold propeller (*32*). The experiments were repeated at least twice with a deviation within ±4%. The binding parameters were obtained by fitting the ITC curves with the model for two binding site sets of identical binding sites.

### SEM and CLSM characterization of bacteria biofilm

The bacteria solution after overnight culture was centrifuged at 7100 rpm for 3 min and washed with PBS twice. After centrifugation, the bacteria were suspended in LB liquid medium, and it was adjusted to OD_600_ = 1.0. Three milliliters of bacteria solution was put into the 12-well plates, which contained carbon paper. The experimental group was added with 20 μM PMNT incubated at 30°C for 12 hours. The carbon paper was washed three times with PBS and fixed with 2.5% glutaraldehyde at 4°C overnight. Then, the glutaraldehyde was removed, and the carbon paper was washed twice with ddH_2_O. The carbon paper was washed with 10, 30, 50, 70, 90, and 100% ethanol for 6 min (*32*). The samples were plated with platinum and examined under a SEM. Three milliliters of bacterial solution was added to the confocal dish. The experimental group was added with 20 μM PMNT, and the control group was not added PMNT incubated at 30°C for 12 hours. Then, the bacterial solution was removed, and the biofilm was washed with PBS once and stained with a LIVE/DEAD BacLight bacterial viability kit according to the instructions. The samples were then detected by CLSM. SYTO 9 was detected by a 488-nm laser, and PI was detected by a 559-nm laser (*32*).

### Electrochemical measurements

Electrochemical measurements were carried out with a standard three-electrode system using Ag/AgCl (saturated in KCl solution) and platinum as the reference and counter electrodes, respectively. The carbon paper from the 12-well plates incubated with bacteria in the absence and presence of PMNT was used as the working electrode. The sealed cell was pumped with nitrogen to remove oxygen. EIS was measured in 1 mM K_4_Fe(CN)_6_ and 0.1 M KCl solution at the open-circuit potential. The frequency range is 10^5^ to 0.1 Hz. CV was measured in PBS from −0.8 to 0.5 V versus Ag/AgCl.

### Electrodeposition of MnO_2_

The electrodeposition was carried out with a standard three-electrode using calomel electrode (saturated in KCl solution), platinum, and carbon paper as the reference, counter, and working electrode, respectively. The electrolyte consists of 0.01 M MnSO_4_ and 0.1 M H_2_SO_4_ using galvanostatic deposition with a current of 5 mA for 1 hour.

### The construction and operation of the detecting system

The bacterial solution after overnight culture was centrifuged at 7100 rpm for 3 min and washed with PBS twice. After centrifugation, the bacteria were suspended in LB liquid medium, and it was adjusted to OD_600_ = 1.0. Four milliliters of bacterial solution was put into the 12-well plates that contained carbon paper. The experimental group was added with 20 μM PMNT incubated at 30°C for 12 hours. Then, the biofilm carbon paper as anode and the MnO_2_ electrode as cathode were put into the sealed cell containing M9 buffer (42 mM Na_2_HPO_4_, 22.0 mM KH_2_PO_4_, 85.5 mM NaCl, and 1.0 mM MgSO_4_), with different concentrations of lactate. The sealed cell was pumped with nitrogen to remove oxygen. The current generated by the system was recorded every 30 min. The system was operated at 30°C. The above test steps were performed independently for three times to operate the detecting system with different concentrations of lactate.

### Lactate testing in cell cultures, sweat, urine, and plasma

All the cells were cultured in Dulbecco’s modified Eagle’s medium (DMEM) with 10% FBS at 37°C in an atmosphere containing 5% CO_2_. The cells were washed twice, counted, and conserved in DMEM (10 × 10^4^, 50 × 10^4^, 100 × 10^4^, 500 × 10^4^, and 1000 × 10^4^). After the cells were cultured for 12 hours, the supernatants of different cells were removed for the detection of lactate concentration through the lactate kit to determine the lactate concentration. The above test steps were performed independently for three times to determine the lactate concentration of different cell number. Besides, the supernatants were added into the sealed cell containing the bioelectronic device to determine the lactate concentration. The above test steps were performed independently for three times to determine the lactate concentration of different supernatants from different cell numbers. Moreover, sweat, urine, and plasma with different lactate concentrations (0, 1, 5, and 10 mM) were added into the sealed cell containing the bioelectronic device to determine the lactate concentration. The above test steps were performed independently for three times to determine the lactate concentration of sweat, urine, and plasma.
