# Supplementary material for: Flexible bioelectronic device fabricated by conductive polymer–based living material
Source: Sci Adv. 2022 Jun 22;8(25):eabo1458. doi: 10.1126/sciadv.abo1458 (PMC9216517; doi:10.1126/sciadv.abo1458)
Supplement: Supplementary file 1 — Figs. S1 to S6 Table S1 [file sciadv.abo1458_sm.pdf]

Science Advances
AAAS

Supplementary Materials for

Flexible bioelectronic device fabricated by conductive polymer–based living material

Zenghao Wang *et al.*

Corresponding author: Shu Wang, wangshu@iccas.ac.cn; Haotian Bai, baihaotian@iccas.ac.cn

Sci. Adv. **8**, eabo1458 (2022)
DOI: 10.1126/sciadv.abo1458

This PDF file includes:

Figs. S1 to S6
Table S1

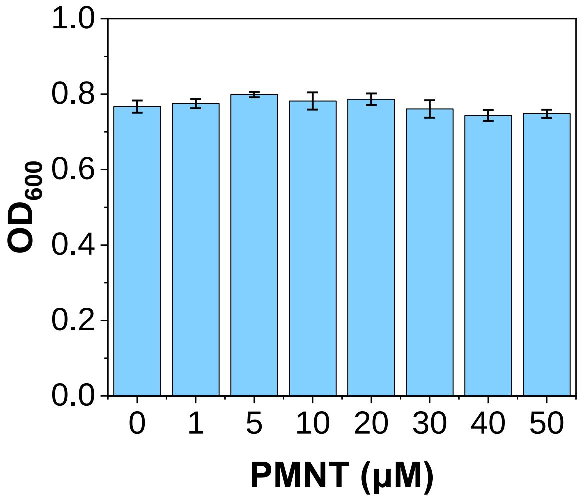

Fig. S1. The OD$_{600}$ of *S. oneidensis* MR-1 with different concentrations of PMNT.

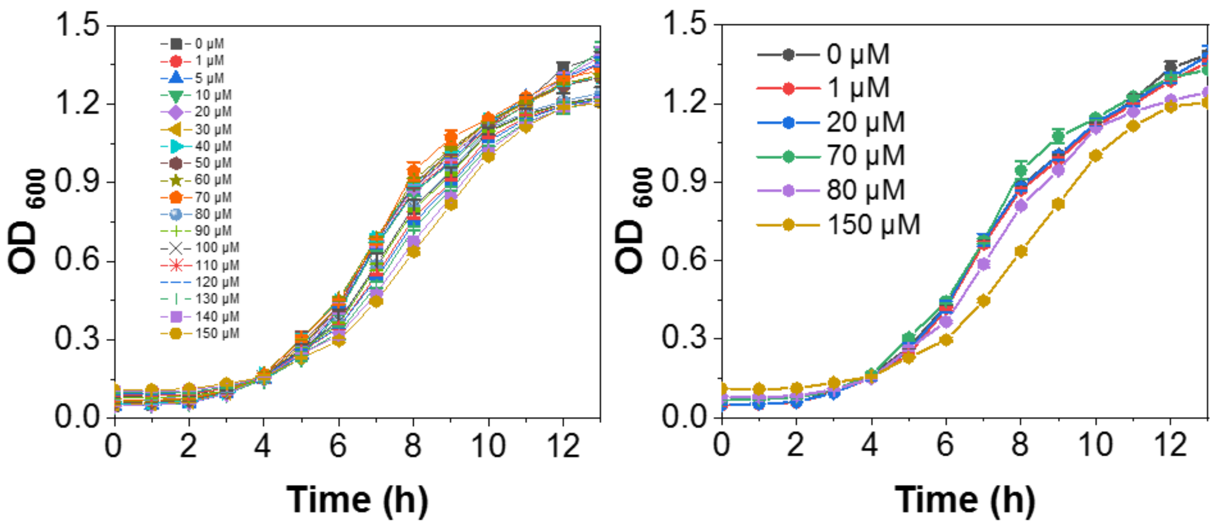

Fig. S2. The growth curves of *S. oneidensis* MR-1 with different concentrations of PMNT. Each line represents the average of three technical replicates with standard error shown in error bars (n=3).

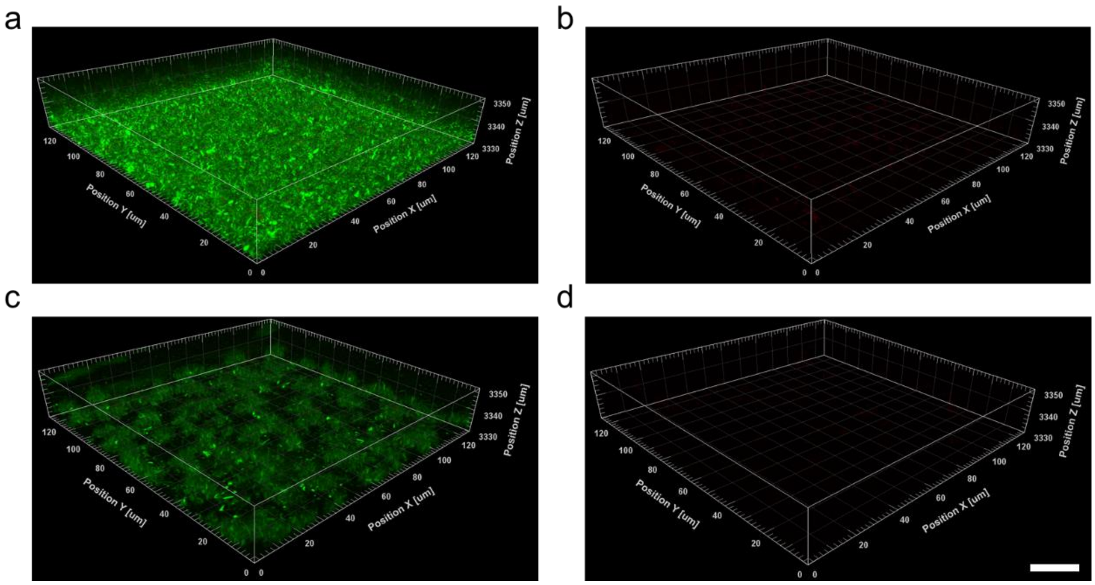

Fig. S3. The CLSM of PMNT-Living material. Merged channels of PMNT/MR-1 (A) and *S. oneidensis* MR-1 (C) biofilm morphology. PI channels of PMNT/MR-1 (B) and *S. oneidensis* MR-1 (D) biofilm morphology, scale bar: 20 μm.

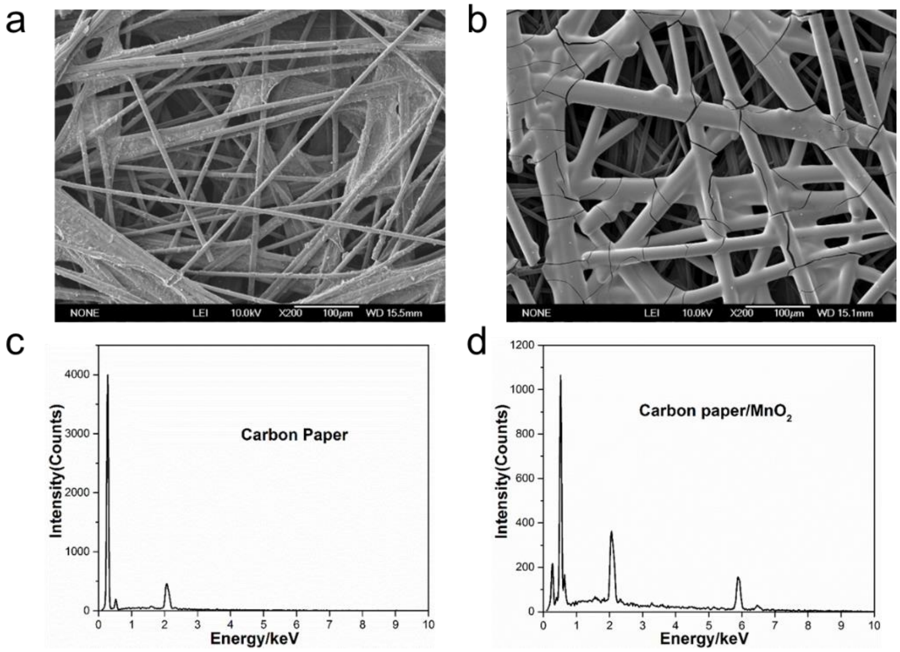

Fig. S4. The characterization of MnO_2. (A) and (B) SEM of carbon paper without and with MnO_2, respectively. (C) and (D) EDS spectrum of carbon paper before and after deposition of MnO_2.

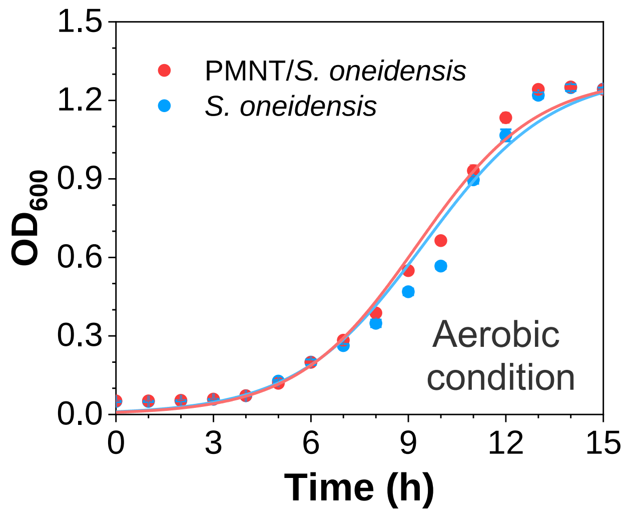

Fig. S5. The growth curves of *S. oneidensis* MR-1 in the absence and presence of PMNT under aerobic condition. Each line represents the average of three technical replicates with standard error shown in error bars (n=3).

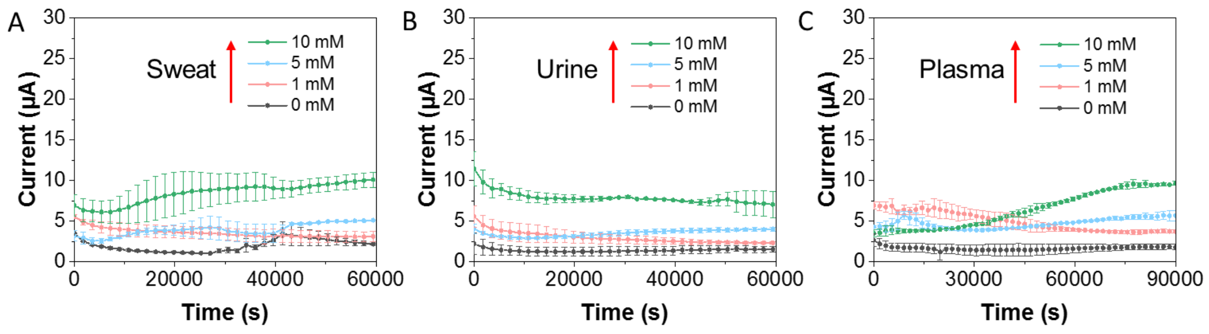

Fig. S6. Current responses generated by the bioelectronic device without PMNT. Current responses generated by the bioelectronic device with the addition of lactate in sweat (A), urine (B) and plasma (C). Each line represents the average of three biological replicates with standard error shown in error bars (n=3).

Table S1. The relative errors of the three cancer cells.

		Cell Line		
	Relative errors	HeLa	MCF-7	A549
Cell Number	1.0×10^6	7.6%	5.6%	7.4%
	5.0×10^6	2.7%	6.8%	9.9%
